# Design Strategies for Optimized Bulk-Linearized MOS Pseudo-Resistor

**DOI:** 10.3390/mi16080941

**Published:** 2025-08-16

**Authors:** Lorenzo Benatti, Tommaso Zanotti, Francesco Maria Puglisi

**Affiliations:** Dipartimento di Ingegneria “Enzo Ferrari”, Università di Modena e Reggio Emilia, Via P. Vivarelli 10/1, 41125 Modena, MO, Italy; lorenzo.benatti@unimore.it (L.B.); tommaso.zanotti@unimore.it (T.Z.)

**Keywords:** pseudo-resistor, bulk linearization, process variation, filter

## Abstract

The bulk linearization technique is a design strategy used to extend the linear region of a metal oxide semiconductor field effect transistor (MOSFET) by increasing its saturation voltage through a composite structure and a gate biasing circuit. This allows us to develop compact and flexible pseudo-resistor elements for integrated circuit designs. In this paper we propose a new simple yet effective design approach, focused on the biasing circuit, that optimizes area, offset, and power consumption without altering the design complexity of the original solution. Post-layout simulations verify the presented design strategy, which is then applied for designing a band-pass filter for neural action potential acquisition. Results of harmonic distortion and noise analysis strengthen the validity of the proposed strategy.

## 1. Introduction

Pseudo-resistors (PSRs) are active components widely used in integrated circuits that require resistors with large values (e.g., from MΩ up to several TΩ), like biomedical acquisition filters [1,2], neural networks [3], and transimpedance amplifiers [4,5]. Depending on the desired specifications (e.g., resistance value, power consumption, area), different circuits have been proposed and developed [6,7,8]. These elements typically allow tuning the resistance value over a wide range, and with significant area savings with respect to their polysilicon counterpart.

Among the different strategies reported in the literature, a recent, simple, and effective implementation of a PSR entails extending the typically limited linear region (<100 mV) of a Metal Oxide Semiconductor Field-Effect Transistor (MOSFET) up to hundreds of mV. This is achieved by means of the so-called bulk linearization (BL) technique [9], which allows tuning the equivalent resistance value for both grounded [9] and floating [10] terminal requirements, even in weak inversion, which is definitely advantageous for ultra-low power applications. Though verified in applications like nano-power low-frequency biomedical acquisition filters [10], operational transconductance amplifiers [11], and true random number generators [12], the recent introduction of this innovative technique still provides an interesting margin for improvement.

**Figure 1:**
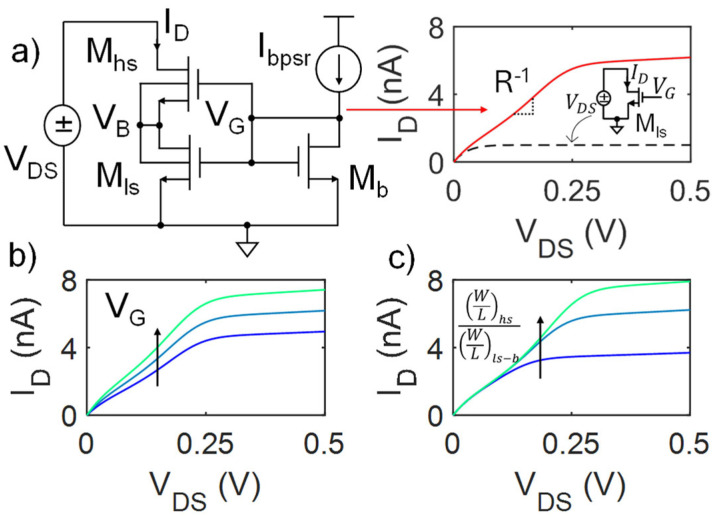
(**a**) BL PSR and its I_D_-V_DS_ characteristic (solid red curve) with a bias current I_bpsr_ through M_b_, compared to a single transistor implementation (black dashed line). Representative PSR characteristics when changing (**b**) V_G_ (by changing I_bpsr_) and (**c**) the aspect ratio (W/L) in the composite transistor M_hs-ls_.

Introduced by Arnaud and Miguez in 2018 [9], the BL technique consists of a composite transistor (M_hs_–high side, and M_ls_–low side, in Figure 1a) with opportunely sized width (W) and length (L), biased to exhibit a specific resistance value, R, in its linear region. M_hs-ls_ (n-MOS in this example) share the same bulk (V_B_) and gate (V_G_) voltage.

Specifically, the bulk terminals are connected to the drain of M_ls_ and to the source of M_hs_. The biasing circuit, providing V_G_ in Figure 1a, is composed of a diode-connected transistor M_b_, equal to M_ls_ [9], driven by a biasing drain current I_bpsr_. If (W/L)_hs_ ≫ (W/L)_ls_, when V_DS_ increases, M_ls_ rapidly saturates, while M_hs_ remains in the linear region. However, as V_B_ increases, V_GS-hs_ decreases to such an extent that I_D_ becomes comparable to the saturation current of M_hs_, finally also driving M_hs_ in saturation. For a fixed transistor size, V_G_ (thus I_bpsr_) defines the resistance of both transistors in the composite transistor, and so the slope of the linear region of the I_D_-V_DS_ characteristic, i.e., R^−1^, as shown in Figure 1b. In particular, increasing (reducing) I_bpsr,_ and so V_G_, reduces (increases) the output resistance of the PSR. This results in a simple, low-power, and tunable PSR. Furthermore, depending on the desired specifications, a wider linear region can be achieved, even in weak inversion, by increasing the asymmetry in the composite transistor, i.e., increasing the ratio between (W/L)_hs_ and (W/L)_ls_, as sketched in Figure 1c. This, in fact, can modulate the voltage drop on M_ls_ and in turn the voltage needed to also drive M_hs_ in saturation. However, especially when the asymmetry is very significant, this strategy can stress the MOSFET non-idealities and lead to less linear output characteristics (e.g., green line in Figure 1c), limiting the exploitation of this technique when high precision is required. Then, as for other approaches, linear regions that are wider, more precise, and more robust to process variations/mismatch (PVM) are achieved by the series connection of multiple BL PSR elements [7,13]. Yet, this directly leads to more area occupation and additional biasing currents, which in turn lead to higher power consumption and current/voltage offset. In particular, as further detailed in Section 2, the offset current in the output characteristic is strongly dependent on the number of BL PSR elements in series, with possible significant deviations from the ideal resistive behavior. Moreover, the conventional BL PSR design is in itself suboptimal and shows margins of improvement in terms of power consumption and robustness to process non-idealities.

Here, we analyze the conventional BL PSR implementation (hereafter, the reference circuit) and its shortcomings. We then propose a new design strategy aimed at optimizing power consumption, current/voltage offsets, PVM robustness, and area, especially when several PSR elements in series are needed. The presented solution is then implemented in a band-pass filter for neural recordings, and its performance is evaluated via post-layout, harmonic distortion, and noise simulations. This paper is organized as follows: Section 2 highlights the issues of the BL technique; Section 3 presents the proposed approach and its advantages; Section 4 illustrates the performance of a filter for neural recording exploiting a PSR designed with the proposed strategy. Conclusions follow.

## 2. Methods

**Figure 2:**
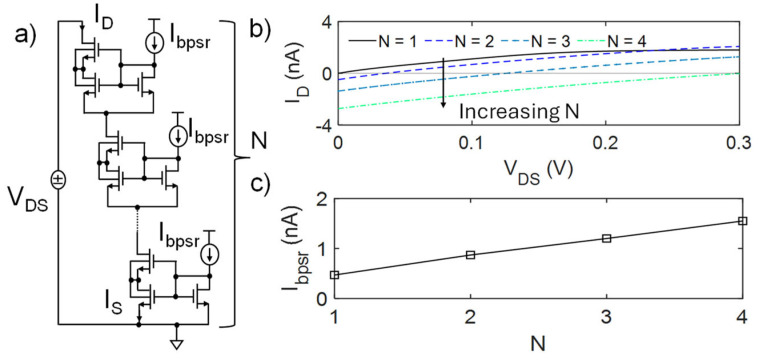
(**a**) PSR composed of N series BL PSR elements and (**b**) the respective I_D_-V_DS_ curve, characterized by an N-dependent offset. (**c**) To achieve the same slope (i.e., R^−1^), each bias current I_bpsr_ must linearly increase with N.

The reference circuit shown in Figure 1a constitutes the basic BL PSR element. As mentioned in the Introduction, the linearity, the extension of the linear range, and the PVM robustness are usually improved by adding multiple independent BL PSR elements in series. Figure 2 shows the series of N PSR elements (Figure 2a) and its N-dependent I-V characteristics (Figure 2b). For a fair comparison, each bias current was set to produce a PSR output characteristic with the same slope (i.e., R^−1^). Nonetheless, the presence of multiple stages and additional bias currents introduced by each element leads to an asymmetry between I_D_ and I_S_, producing an offset current (I_D_ at V_DS_ = 0, i.e., I_osN_) and voltage (V_DS_ at I_D_ = 0, i.e., V_osN_) that depend on N and that can be empirically expressed as follows:

(1) $$I_{os_{N}}={I_{D}}_{N}\left(V_{DS}=0,\;N\geq 2\right)≅\frac{2-N^{2}}{2N}{\cdot I}_{b_{psr}}$$

(2) $$V_{os_{N}}=R\cdot |{I_{os}}_{N}|$$

**Figure 3:**
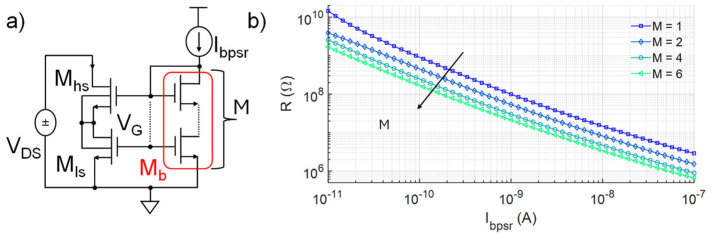
(**a**) Proposed implementation of the BL PSR element. (**b**) Resistance value R vs. I_bpsr_ relation for different values of M (N = 1, m = 1).

This effect is due to the fact that the biasing current of each element sums up to its input current at the source terminal of the transistors at the bottom of the element, so that the input current of the element below is increased, determining an imbalance and, thus, the offset current and voltage. Such offsets can be compensated for or regulated by opportunely matched sink current sources placed at the gate of each PSR element or through additional offset current sources [9]. Yet, this leads to increased design complexity, area occupation, and PVM sensitivity. Also, to achieve the same slope (i.e., R**^−^**^1^), the value of each I_bpsr_ current source should linearly increase with N in order to reduce the resistance value of each element, as reported in Figure 2c, with an obvious impact on power consumption of the PSR element and of the circuit in which it will be included. To tackle these challenges, the proposed design strategy starts from modifying the bias part by decoupling M_ls_ and M_b_ shape factors (W/L)_ls_ and (W/L)_b_, without altering those of the composite transistor M_hs-ls_, i.e., the core of the pseudo-resistor. Depicted in Figure 3a, the proposed approach consists of achieving the same bias voltage V_G_ of the reference circuit but with (W/L)_b_ < (W/L)_ls_. This introduces a further asymmetry in the circuit, which can be exploited to overcome the aforesaid limitations and optimize the overall design. Although applicable for different shape factors, for clarity’s sake, in the following description, (W/L)_ls_ was set to the minimum value allowed for a single transistor, (W/L)_hs_ to the maximum, and M_b_ was considered as composed by the series of M transistors equal to M_ls_, hence,

(3) $${\left(\frac{W}{L}\right)}_{b}=\frac{1}{M}{\left(\frac{W}{L}\right)}_{ls}$$

In the following paragraphs, the advantages of this design strategy were discussed considering N as the number of series PSR elements, M as the number of series transistors in the bias part (i.e., M_b_), and m as the number of parallel transistors for M_hs_, responsible for the linear region extension of the single PSR element. Note that the reference circuit corresponds to the case M = 1. Although a wide range of R values can be obtained depending on the chosen I_bpsr_, as shown in Figure 3b, for simplicity the analysis is carried out for the intermediate and representative case of R = 100 MΩ. Also, given the typical series implementation employed in PSR circuits and to achieve a linear range of at least 200 mV, N ≥ 2 will be considered hereafter. The following examples were implemented in the IHP SG13S 130 nm BiCMOS technology, employing high-threshold voltage MOS transistors (nmosHV) operating in weak inversion, with minimum and maximum size for a single transistor equal to 330 nm and 10 µm, respectively. Thus, the reference circuit, with M = 1, is characterized by (W/L)_ls-b_ = 0.33 µm/10 µm and (W/L)_hs_ = 10 µm/0.33 µm. Post-layout simulations were carried out considering a reference temperature of 27 °C.

## 3. Results

### 3.1. Power Consumption

Without considering the output branch, the current and power consumption of which depend on the desired resistance and applied voltage, the only contributor to the power consumption of the BL PSR element is its biasing current I_bpsr_. Reducing the effective shape factor of the biasing transistor M_b_ by increasing M immediately leads to a lower bias current I_bpsr_ for achieving the same bias voltage V_G_, so as to achieve the same R (as in Figure 3b). In particular, since M_b_ works in the saturated weak inversion region [14,15],

(4) $$I_{b_{psr}}={\left(\frac{W}{L}\right)}_{b}I_{0}e^{\frac{V_{GS}-V_{TH}}{nV_{t}}}(1+\lambda V_{DS})$$

(5) $$V_{GS}=V_{G}≅nV_{t}\ln\left(\frac{{I_{b}}_{psr}}{I_{0}{\left(\frac{W}{L}\right)}_{b}}\right)+V_{TH}$$

where I_0_ considers transistor technology property, such as oxide capacitance, carriers’ concentration, and mobility. V_GS_ is the gate–source voltage, V_t_ is the thermal voltage, V_TH_ is the threshold voltage, *n* the subthreshold slope factor and λ the channel length modulation coefficient, a technology-dependent parameter describing a residual dependence of the drain current on V_DS_ even when the MOSFET is in saturation, identified as the inverse slope of the I_D_-V_DS_ plot in saturation and typically in the range from 0.01 to 0.1 V^−1^ or even higher, depending on the specific MOSFET and its operating condition. Recalling (3), to maintain the same V_G_, I_bpsr_ should scale as 1/M.

(6) $$I_{bpsr}=\frac{I_{bpsr(M=1)}}{M}$$

**Figure 4:**
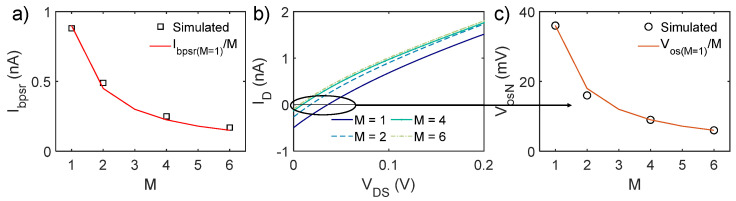
(**a**) I_bpsr_ scaling for different M in order to achieve the same bias voltage V_G_, hence resistance value R. (**b**) I_D_-V_DS_ characteristics for different M values. (**c**) The associated voltage offset. In all cases, N = 2.

This is verified in Figure 4a, where I_bpsr_ is shown to scale as expected, with beneficial consequences on the overall energy efficiency. For N = 2, I_bpsr_ currents are set from 880 pA (M = 1) to 150 pA (M = 6), in order to achieve the same V_G_ = 525 mV in the bottom PSR element. The upper stage shows a maximum V_G_ of 650 mV for V_DS_ = 0.2 V. For further power consumption calculations, it is assumed that I_bpsr_ currents are driven by p-MOS current mirrors with a source voltage (i.e., supply voltage) of 1 V in order to provide sufficient voltage headroom for the p-MOS transistors to work in saturation and to strongly suppress the sensitivity to supply voltage variations.

### 3.2. Offset Reduction

Recalling Equations (1) and (2), offset current and voltage have a direct dependence on I_bpsr_, the number N of series PSR elements, and the target R value. Reducing I_bpsr_ is then crucial in order to keep the offset under control and to restore a resistive behavior closer to the ideal one, especially when the design requires high precision and PVM robustness. The effect of M series MOSFETs equal to M_ls_ at the bias side is reported in Figure 4 for N = 2. Figure 4b shows the I_D_-V_DS_ characteristics of structures with different M, while Figure 4c shows V_os_ for R = 100 MΩ. As expected, V_os_ scales as I_bpsr_, shifting from almost 40 mV when M = 1 (i.e., the reference circuit) to 6 mV when M = 6.

### 3.3. Process Variations/Mismatch

**Figure 5:**
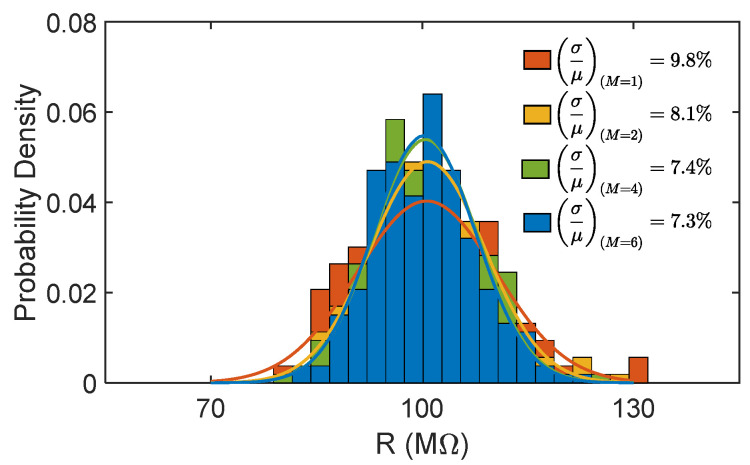
Distributions of the resistance value R obtained with 200 Monte Carlo runs including PVM at different values of M, with N = 2.

The biasing side of the analyzed PSR element also plays a crucial role in determining the robustness of the entire circuit (i.e., series of N PSR elements) to PVM. In fact, especially for circuits in weak inversion, increasing the area of the transistors remains a necessary strategy to keep PVM sensitivity under control, due to the exponential relation between I_D_ and V_TH_. The insertion of additional series transistors then results in an increase in the area of the effective biasing transistor M_b_ but with direct benefits on the circuit variability. PVM sensitivity can be roughly expected to scale as 1/√M [16]. The results of 200 Monte Carlo runs are reported in Figure 5 for the representative case with N = 2, m = 1, and M varying from 1 (reference circuit, purple) to 6 (yellow). Considering the case with M = 4 as the optimal one (for M = 6 the improvement compared to M = 4 is almost negligible), the ratio between the standard deviation (σ) and the mean value (µ) of the R distribution drops from 9.8% to 7.4% with just a 10% area overhead in the single PSR element.

### 3.4. Area Occupation

**Figure 6:**
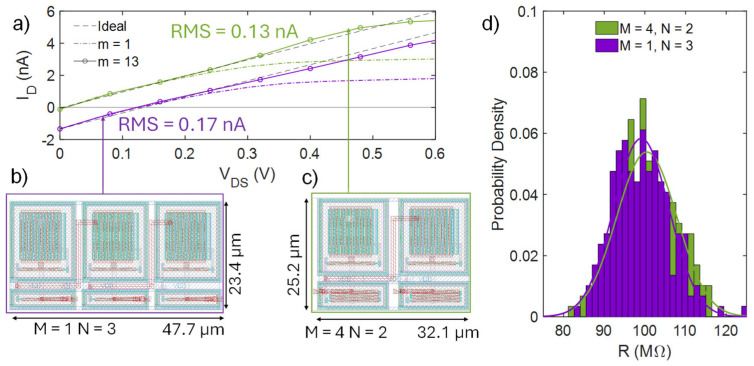
Post-layout comparison of two BL PSRs (M = 4 and N = 2 (cyan), M = 1, and N = 3 (reference, purple) sized for a 100 MΩ^-1^ slope and similar linear region extensions (500 mV): (**a**) Output characteristics for m = 1 (dot-dashed lines) and m = 13 (solid lines with symbols), compared with the ideal resistive behavior (black dashed lines). (**b**,**c**) Layout of the analyzed circuits (m = 13) and (**d**) PVM analysis via 200 Monte Carlo simulation runs.

For typical use cases where multiple PSR elements are stacked in series to achieve high saturation voltages, the proposed solution (M > 1) not only reduces power consumption but also provides significant area savings compared to the implementation where each PRS element is realized with a reference circuit (M = 1), in spite of the small area overhead introduced at the single BL PSR element level, while maintaining or even improving other PSR metrics. Figure 6a shows the I_D_-V_DS_ characteristics of a PSR composed of the series connection of three BL PSR elements (N = 3), with M = 1, designed to have R = 100 MΩ and a linear range extension of 0.5 V. This extension is achieved with m = 13. The same linear range and R are also obtained using an implementation with M = 4 and N = 2, keeping m = 13.

With the latter implementation, the root mean square (RMS) error with respect to an ideal linear behavior improves from 0.17 nA to 0.13 nA. Figure 6b,c show the layout of these two circuits. Remarkably, both circuits exhibit a PVM sensitivity (σ/µ) around 7.4%, as shown in Figure 6d. The introduced additional asymmetry in each individual PSR element (M > 1) allows achieving the same performances as in the implementation with M = 1, together with the following emerging benefits:

- The sum of the bias current drops from 3.6 nA to 400 pA (−89%), since now each PSR element should exhibit a higher R (lower I_bpsr_) to keep the overall R unchanged;
- The offset voltage drops from 120 mV to 9 mV (−92.5%) because of the lower I_bpsr_;
- The total area occupation drops from 1116 µm^2^ to 809 µm^2^ (−27.5%) since fewer PSR elements are employed;
- The −3 dB bandwidth (BW) increases from 6 kHz to 10.5 kHz (+75%).

The first two columns of Table 1 summarize and compare the properties of the two implementations in Figure 6. Although the R range could be further extended toward higher R values by decreasing I_bpsr_, we report R_max_ = 1 GΩ as the maximum resistance value as this corresponds to I_bpsr_ = 10 pA, close to what is employed in [8]. It has to be noted that these two designs, i.e., (M = 4, N = 2) and (M = 1, N = 3), could each be changed by trading off area vs. PVM sensitivity. In the second column of Table 1, besides reporting the performance of the design with M = 1 and N = 3, we also include the results for a design in which M = 1 and N = 2 (while keeping R = 100 MΩ), which guarantees a slight improvement in terms of area consumption compared to the proposed design but at the cost of increased PVM sensitivity (and both worse linearity and worse linear region extension). Then, to better appreciate both the advantages of the BL PSR vs. other alternatives in the literature and the improvements brought about by the proposed design strategies, a specific figure of merit (FOM) has been included in the table: FOM = σ/μ⋅√A. As increasing the area (A) directly benefits PVM sensitivity (proportionally to √A), this FOM allows a direct comparison of the extent to which each solution can optimize the area/PVM sensitivity trade-off. The proposed design also achieves best-in-class FOM and power consumption when compared to non-BL PSR designs, resulting in an extremely efficient PSR over a wide range of possible resistance values.

## 4. Discussion

To test the validity of the proposed approach, the PSR is now employed in an exemplificative band-pass filter designed for neural action potential acquisition, exploiting the same 100 MΩ PSR designs in Figure 6. To obtain more insights, the analysis is extended to designs with different values of M and N, keeping m = 13. This application, which is similar to the one used to test the performance of the BL PSR in [9], represents a valuable use case for investigating any possible further advantage or drawback that the proposed design strategy brings compared to the reference one. The filter is designed for a 20 dB gain in the 500 Hz–8 kHz band, required for action potential recording [6,17], and V_DD_ = 1 V.

**Figure 7:**
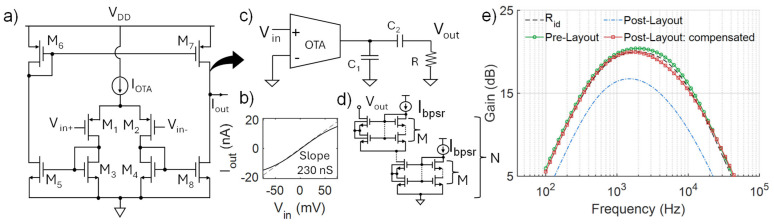
(**a**) Schematic of the implemented p-MOS input OTA (Gm cell) and (**b**) its output curve. (**c**) Analyzed band-pass filter, with (**d**) the proposed bulk-linearized pseudo-resistor with different M and N values. (**e**) Filter frequency response with ideal R_id_ = 100 MΩ (black dashed line), and PSR (M = 4, N = 2, m = 13). Pre-layout (green circles) and post-layout simulations (blue dash-dotted line) are reported. The nominal behavior can be effectively restored by choosing C_1_ = 200 fF and C_2_ = 700 fF (red squares).

To boost the gain but also fulfill the typical low-power requirements of this circuit class, the first stage of the filter is realized with a low-current p-MOS input operational transconductance amplifier (OTA) with a bias current I_OTA_ = 20 nA, as shown in Figure 7a. Except for M_5_ and M_8_, all the transistors are sized with (W/L) = 10 µm/5 µm. Without changing the bias current, the output conductance (I_out_ vs. V_in_ slope in Figure 7b) can be finely tuned by adjusting the shape factor of M_5_ and M_8_, responsible for the output characteristics. The OTA transconductance value of 230 nS in Figure 7b is obtained with (W/L)_5–8_ = 10 µm/4.4 µm. Given the typical amplitude of the signals to be acquired (e.g., tens to hundreds of µV [18,19]), the linearity of the transimpedance cell only needs to be verified over a relatively small input span (i.e., ±50 mV). Finally, to set the bandwidth in the 500 Hz–8 kHz range required for typical neural signal acquisition [6,17], C_1_ and C_2_ in Figure 7c are set to 500 fF.

Figure 7e reports the ideal behavior (black dashed line) of the designed filter, obtained with an ideal resistor, compared to the implementations using the PSRs. For simplicity, only the case M = 4 is reported, although all the different implementations tested in this work show comparable results. While the nominal case (pre-layout) of this circuit leads to an almost ideal response (green circle), post-layout simulations (blue dot-dashed line) show significant deviations from the ideal behavior. This is mainly due to the process requirements of the BL technique, which are shared with other designs and techniques as well [5]. Specifically, the need for an isolated bulk connection for the M_hs-ls_ composite transistor inevitably translates to the need for a triple-well, with the introduction of an additional capacitive contribution due to the p-n junction of the well, independently of the adoption of the proposed strategy (i.e., this also applies to the reference circuit). Yet, this can be effectively compensated for by changing the values of C_1_ and C_2_ (to 200 fF and 700 fF, respectively), restoring the nominal behavior (Figure 7e).

**Figure 8:**
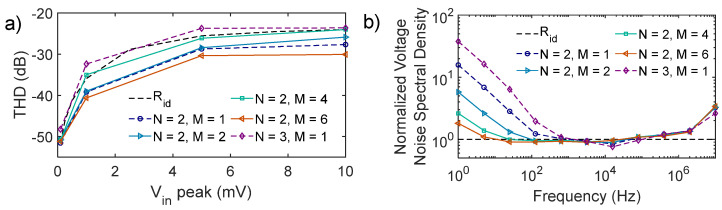
(**a**) THD values of the filter for different M and N in the bulk-linearized PSR. Dashed lines are for implementations using the reference circuit (M = 1). The results obtained when employing an ideal resistor, R_id_ = 100 MΩ, are reported with a black dashed line. (**b**) Voltage noise spectral density vs. frequency behavior of the filter with different PSRs implementations. Results are normalized with respect to the filter with an ideal resistor, R_id_ = 100 MΩ (black dashed line).

The filter is now studied in terms of total harmonic distortion (THD). Figure 8a illustrates the THD for different small signal input peak amplitudes. Specifically, results for the reference circuit (M = 1) for N = 2 and N = 3 are reported with dashed lines, while those for the proposed design are reported with continuous lines. The analysis highlights that reducing the shape factor of the biasing transistor M_b_ while increasing its area brings no THD degradation, in fact resulting in a marginal additional benefit, especially for M = 6. Results are in line with the THD levels obtained when employing an ideal resistor R_id_ = 100 MΩ (black dashed line), which further highlights that the main contributor to THD is the OTA, the optimal design of which is beyond the scope of this paper.

Recalling the analysis performed in the previous section, given a 1400 µm^2^ footprint for the OTA, the adoption of the PSR with M = 4, N = 2, and m = 13 (Figure 6b) brings an overall 12% area savings for the filter under consideration with respect to the case with M = 1, N = 3, and m = 13 (Figure 6c), together with a significant offset voltage reduction, same PVM sensitivity, and reduced power consumption.

The results of voltage noise spectral density simulations, normalized with respect to the filter implementation with an ideal resistor (i.e., R_id_ in Figure 8b), additionally remark that leveraging the asymmetry between M_b_ and M_ls_ brings further benefits, especially at frequencies below 1 kHz.

## 5. Conclusions

In this work, we introduced and verified a design technique for the optimization of a BL PSR. Reducing the shape factor while increasing the area of the biasing transistor introduces an additional asymmetry in the circuit that is leveraged to significantly improve power consumption, offset, PVM robustness, BW, and area occupation, specifically when strong linearity is required over a wide voltage range. This results in an extremely competitive design compared to other reports. The proposed design was integrated in a band-pass filter for neural action potential acquisition. Post-layout simulations show no THD degradation, noise improvement, and 12% area savings at lower power consumption and similar PVM sensitivity and linear range.

## Figures and Tables

**Table 1:** For a fair comparison, the area occupation and power consumption are reported for the PSR elements only, without considering the impact of the circuitry implementing the bias current sources.

	This Work ^a^	[9] ^a^		[5] ^b^	[8] ^b^	[7] ^b^	[6] ^b^
BL	yes	Yes		no	No	No	No
Tech. (nm)	130	130		180-SOI	180	350	350
R_min_	1 MΩ	1 MΩ		1 MΩ	180 GΩ	20 MΩ	500 MΩ
R_max_	1 GΩ	1 GΩ		1 GΩ	700 GΩ	20 GΩ	70 GΩ
BW @R_min_	1.3 MHz	380 kHz		2 MHz	100 Hz	10 MHz	8 kHz
BW @R_max_	1.3 kHz	650 Hz		8 kHz	3 Hz	100 kHz	0
Supply	1 V	1 V		1.8 V	1.8 V	3.3 V	3.3 V
Power (nW)	N = 2	N = 2	N = 3	200k	5.4	100	2000
	0.4 *	2 **	3.6 **				
Offset (mV)	9 *	36 **	120 **	-	-	-	-
σ/µ (%)	7.4 *	9.8 **	7.4 **	3.6	2.53	25.5	13
Area (µm^2^)	809 *	730 **	1116 **	16k	16.5k	17.7k	54k
FOM	2.1 *	2.6 **	2.5 **	4.6	3.2	34	30

* M = 4 and N = 2, R = 100 MΩ. ** M = 1, R = 100 MΩ. The design in [9] is simulated using the same technology as in this work for a direct comparison with the proposed design strategy. ^a^ Post-layout. ^b^ Measured.

## Data Availability

The original contributions presented in this study are included in the article. Further inquiries can be directed to the corresponding author.

## Abbreviations

The following abbreviations are used in this manuscript:

BL	Bulk-linearized/Bulk Linearization
PSR	Pseudo-resistor
MOSFET	Metal Oxide Semiconductor Field Effect Transistor
PVM	Process/Variation Mismatch
OTA	Operational Transconductance Amplifier
FOM	Figure of Merit
THD	Total Harmonic Distortion
